# Impact of High-Pressure Processing (HPP) on *Listeria monocytogenes*—An Overview of Challenges and Responses

**DOI:** 10.3390/foods13010014

**Published:** 2023-12-19

**Authors:** Patryk Wiśniewski, Wioleta Chajęcka-Wierzchowska, Anna Zadernowska

**Affiliations:** Department of Food Microbiology, Meat Technology and Chemistry, Faculty of Food Science, University of Warmia and Mazury, Plac Cieszyński 1, 10-726 Olsztyn, Poland; wioleta.chajecka@uwm.edu.pl (W.C.-W.); anna.zadernowska@uwm.edu.pl (A.Z.)

**Keywords:** high-pressure processing, pascalisation, foodborne pathogens, non-thermal, *Listeria monocytogenes*

## Abstract

High-pressure processing (HPP) is currently one of the leading methods of non-thermal food preservation as an alternative to traditional methods based on thermal processing. The application of HPP involves the simultaneous action of a combination of several factors—pressure values (100–600 MPa), time of operation (a few–several minutes), and temperature of operation (room temperature or lower)—using a liquid medium responsible for pressure transfer. The combination of these three factors results in the inactivation of microorganisms, thus extending food shelf life and improving the food’s microbiological safety. HPP can provide high value for the sensory and quality characteristics of products and reduce the population of pathogenic microorganisms such as *L. monocytogenes* to the required safety level. Nevertheless, the technology is not without impact on the cellular response of pathogens. *L. monocytogenes* cells surviving the HPP treatment may have multiple damages, which may impact the activation of mechanisms involved in the repair of cellular damage, increased virulence, or antibiotic resistance, as well as an increased expression of genes encoding pathogenicity and antibiotic resistance. This review has demonstrated that HPP is a technology that can reduce *L. monocytogenes* cells to below detection levels, thus indicating the potential to provide the desired level of safety. However, problems have been noted related to the possibilities of cell recovery during storage and changes in virulence and antibiotic resistance due to the activation of gene expression mechanisms, and the lack of a sufficient number of studies explaining these changes has been reported.

## 1. Introduction

High-pressure technology (HPP) is a low-temperature treatment of food to improve its microbiological safety by inactivating pathogenic microorganisms while minimally affecting the nutritional, functional, and/or sensory properties of food products undergoing preservation [1,2]. The technology is applied to products where other food processing technologies (including heat treatment) negatively impact their quality and appearance relative to the raw material [3]. According to industry data, the use of HPP technology is increasing in both the US and EU countries [4]. One of the main drawbacks limiting the widespread availability of HPP equipment is its high cost, ranging from EUR 500,000 to EUR 3,500,000 [5]. Currently, a variety of HPP products such as fruit juices, packaged vegetables, meat, and RTE meat products, as well as dairy products and seafood, are available at retail [6,7,8,9]. During fixation using HPP, the most common pressures used are 100–600 MPa, with different operating times (a few–several minutes) at room temperature (or lower), in flexible packaging, using a liquid medium responsible for pressure transfer [10].

One of the most dangerous food pathogens isolated from various foods is *Listeria monocytogenes* [11]. Due to its virulence and ability to spread in the environment, *L. monocytogenes* is one of the main threats to safe food production [12]. The extent of induced changes, damage, and/or cell death of *L. monocytogenes* under the influence of HPP technology depends on several factors, among which are the parameters of the process (pressure value, time of treatment), the type of food matrix, and the presence of antibacterial compounds [9]. Some of the injuries caused by HPP technology within cells may be reversible during the storage of food products. This can affect the re-growth of microorganisms, resulting in food spoilage or the induction of foodborne diseases [13].

Environmental stress induced by HPP directly affects changes not only in the pathogen’s cell structure, but also within the cell’s genome, which consequently translates into changes in the cell’s pathogenicity—including changes in virulence, antibiotic resistance, and the expression of genes encoding these resistances [10,14,15]. There are few studies on the effect of HPP treatment-dependent factors (pressure, operating time, temperature) and food matrix (water activity, pH, moisture content, fat content, NaCl content) on reducing *L. monocytogenes* in various food products [16,17,18]. Most studies on HPP treatment focus on the effectiveness of HPP against pathogen cell inactivation while maintaining food product quality [19]. This review highlights the lack of sufficient studies explaining antibiotic resistance, virulence, and the expression of antibiotic resistance and virulence genes in *L. monocytogenes* cells that survive HPP treatment, as well as explaining the potential for *L. monocytogenes* cell recovery during storage. Knowledge related to both the pathogen itself and the impact of HPP on *L. monocytogenes* should continue to expand to improve industrial applications of HPP treatment, inactivate *L. monocytogenes* and, consequently, produce safe food.

## 2. *Listeria monocytogenes*—A Virulent, Psychrotrophic Foodborne Pathogen

The occurrence of pathogenic microorganisms in food is well-known. Through their occurrence in food, they can contribute to causing various foodborne diseases [20]. Among the group of the most dangerous pathogens that pose a threat to public health and the food industry is *L. monocytogenes* [21]. These microorganisms are isolated from water, soil, wastewater, vegetation, fish, birds, and mammals, and thus, also from food [22,23]. Their ability to survive adverse environmental conditions is determined by the ubiquity of *L. monocytogenes* in the environment. Food is considered the main source of *L. monocytogenes*—including, for example, raw and smoked fish, meat products, unpasteurized dairy products, fruits, vegetables (including frozen vegetables), seeds, and spices, as well as ready-to-eat (RTE) products [24].

*L. monocytogenes* is a heterogeneous species comprising 13 serotypes, which can be divided into four genetic groups. Serotypes 1/2b, 3b, 4b, 4d, and 4e belong to group I, while serotypes 1/2a, 1/2c, 3a, and 3c are included in group II. Serotypes 4a and 4c belong to group III and are rarely isolated from humans. Serotypes 1/2a and 1/2b are most often isolated from food, while 4b is isolated from clinical cases [25]. According to European Commission Regulation (EC) No. 2073/15 November 2005 [26], *L. monocytogenes* is considered one of the criteria for food safety. Its occurrence should be monitored in products where its growth is possible before these products enter the market. In such products, *L. monocytogenes* should not be present in 25 g (25 mL) of a sample. Additionally, throughout a product’s shelf life, the number of these bacteria in a sample must not exceed 100 CFU/g (CFU/mL) of the product, regardless of whether the product favours or does not favour the growth of these bacteria. Food testing is usually limited to detecting the presence or determining the number of this pathogen, and omits testing for, among other things, determining growth after storage, which is particularly important because of the possibilities associated with the growth of *L. monocytogenes* during storage.

### 2.1. Listeria monocytogenes—Virulence Factors

*L. monocytogenes* has a whole range of mechanisms that directly affect its pathogenicity (virulence). The pathogenicity mechanism of *L. monocytogenes* is a complex process with two main phases: (I) the intestinal phase that involves the initial bacterial colonisation in the intestine and the penetration through the mucosal barrier into the bloodstream or the lymphatic system in order to spread throughout the body; and (II) the systemic spread phase, during which dendritic cells or macrophages transport bacterial cells to the spleen, lymph nodes, the liver, the brain, and the placenta (in pregnant women) [27]. A variety of virulence factors are involved in particular stages of the pathogenesis process, including protein regulatory factor (PrfA), sigma B factors (σ^B^), adhesion proteins, epithelial cell invasion factors, factors responsible for vacuole lysis, factors responsible for cell-to-cell spread, and other unclassified factors (Table 1) [27,28].

The various virulence factors are encoded by their corresponding genes, and their role in the pathogenicity process depends on their expression [29]. To date, four pathogenicity islands have been identified, defined as Listeria Pathogenicity Islands (LIPIs). The typical genetic organization of LIPIs includes the islands LIPI-1, LIPI-2, LIPI-3, and LIPI-4 [29]. Each island encodes a different set of genes. LIPI-1 consists of six genes (*prfA*, *actA*, *hly*, *mpl*, *iap*, *plcA, and plcB*) and is essential for intracellular survival and cell proliferation. LIPI-2 is a 22 kb cluster of genes responsible for phagosome degradation. LIPI-3 consists of eight genes (*llsA*, *llsG*, *llsH*, *llsX*, *llsB*, *llsY*, *llsD*, *llsP*) encoding a biosynthetic cluster involved in Listeriolysin S (LLS) production. The last, LIPI-4, is a cluster that includes six genes (*GlvA*, *Gat-pr*, *YdjC*, *GatA*, *GatB*, *GatC*) involved in neuronal and placental infection [30,31,32,33,34]. 

In addition to the presence of different virulence-encoding genes, ongoing research indicates that *L. monocytogenes* possesses mechanisms for surviving adverse environmental conditions. One such mechanism is the ability of *L. monocytogenes* to enter a viable but non-culturable (VBNC) state in which the cells retain their biological activity. Nutrient loss has been demonstrated to be the primary trigger for cultured cells to enter the VBNC state [35,36]. However, salinity, temperature change, low environmental pH, chlorine-induced stress, or exposure to sunlight may also play an important role in triggering the VBNC state in *L. monocytogenes* [37,38,39]. Pathogenic microorganisms in the VBNC state may pose a potential hazard to food safety due to the retention of their cellular integrity and ability to express genes, which in many cases is much stronger than that of cultured cells [40]. There is, therefore, a risk that *L. monocytogenes* bacteria will not be detected at the production facility when using traditional microbial culture techniques and will be present in the VBNC state in food, with the result that the pathogen levels will increase and may contribute to causing diseases in people consuming such contaminated food. It is therefore necessary to undertake new research in order to understand the actual risk associated with the occurrence of *L. monocytogenes* in the VBNC state in RTE-type foods, as well as the virulence of this pathogen [21].

### 2.2. Listeriosis

*L. monocytogenes* is responsible for causing listeriosis—a zoonosis characterized by a very severe course, especially in people in the so-called high-risk group, which includes the elderly, pregnant women, newborns, and immunocompromised people [25]. Listeriosis is characterized by a severe course (meningitis, endocarditis and encephalitis, sepsis, miscarriages, stillbirths) and a high mortality rate of 20–30% [41,42]. 

In the treatment of listeriosis, antibiotics are used to inhibit the infection caused by *L. monocytogenes*. This bacterium is considered sensitive to a broad spectrum of antibiotics that exhibit bactericidal activity against Gram-positive bacteria—including tetracyclines, ampicillin, penicillin G, imipenem, amoxicillin, sulphonamides, aminoglycosides, macrolides, chloramphenicol, and glycopeptides [43]. Despite this, most *L. monocytogenes* strains show native resistance to cefotaxime, cefepime, fosfomycin, oxacillin, and lincosamides [44]. In the literature, information is encountered regarding the occurrence of natural resistance to fosfomycin, third-generation cephalosporins, and first-generation quinolones [45]. Resistance to tetracyclines is considered the most common antibiotic resistance trait in *L. monocytogenes* isolated from humans and food. Six classes of tetracycline resistance genes (*tet*K, *tet*L, *tet*M, *tet*O, *tet*P, and *tet*S) have been described in Gram-positive bacteria. However, only *tet*S, *tet*M, and *tet*L have been identified in *L. monocytogenes*. The study also found that strains isolated from dairy farms had more than one antibiotic resistance gene sequence in their genome. A high frequency of the *flo*R (66%), *pen*A (37%), *str*A (34%), *tet*A (32%), and *sul*I (16%) genes was found in a large number of *L. monocytogenes* strains isolated from food, although the other tetracycline resistance genes (*tet*E, *tet*C, *tet*B, *tet*D, and *tet*G) and other antibiotic resistance genes (*van*A, *van*B, *aad*A, *cml*A, *ere*B, *ere*A, *str*B, *sul*I, *amp*C, and *erm*B) were not detected in *L. monocytogenes* [44,46]. Researchers report that *L. monocytogenes* isolated from different foods show the presence of the following genes: *tet*M [47]; *erm*B, *tet*M, *dfr*D [48], *tet*A, *lmr*B, *mec*C, *msr*A, and *fos*X [49]; *erm*B [50]; and *Lde*, *aadB*, *mefA*, *lnuA*, *sulI*, and *sulII* [11]. Resistance to tetracyclines and other antibiotics in *L. monocytogenes* is mainly caused by conjugation plasmids and transposons from *Enterococcus* sp., *Streptococcus* sp., and other *Listeria* sp. [44]. Although most strains within a species show high susceptibility to antibiotics, the massive use of antibiotics in human and animal medicine leads to the exposure of microbial strains to sub-therapeutic concentrations of antibiotics [51], which promotes the development of resistance in these pathogens and the activation of stress response mechanisms, including the activation of “silent” antibiotic resistance genes. Cross-resistance has also been observed in *L. monocytogenes*, with the result that the strains that have developed resistance to benzalkonium chloride (a component of disinfectants often used in the food industry) are also resistant to ciprofloxacin due to the efflux pump activity [52,53]. *L. monocytogenes* poses an ongoing threat to the food industry, particularly at facilities producing RTE-type foods [11]. In recent years, there has been a steady increase in antibiotic resistance among *L. monocytogenes* strains isolated from food and food production environments (especially for the antibiotics used to treat listeriosis), and it is, therefore, necessary to monitor changes with regard to the antibiotic resistance of *L. monocytogene*s strains isolated from food and the emergence of multi-resistant strains [11,44,51,54,55,56,57,58,59,60,61,62].

### 2.3. Food-Related Outbreaks Caused by Listeria monocytogenes

Listeriosis was the fifth most frequently reported zoonotic disease in humans in the EU in 2021. The prevalence of *L. monocytogenes* was different depending on the food category and sampling stage. Overall, the prevalence remained at a low level in RTE foods. The highest values were observed for fish and fishery products (3.5–5.4%), meat products of beef or pork origin (2.7–3.9%), fruits and vegetables (2.5%), and hard cheeses made from raw or low-heat-treated sheep’s milk (4.6%). As in previous years, the highest percentage of positive samples for *L. monocytogenes* was observed at the production stage compared to the distribution stage [63].

Recently, many listeriosis outbreaks have been transmitted by many different types of food worldwide—in Europe [64,65,66,67,68], USA [69,70,71,72,73,74,75,76,77,78,79,80,81,82,83,84] Africa [85], and Australia [86] (Table 2). The most recently documented case of an outbreak of listeriosis took place in two US states, namely Florida and Ohio, and was associated with the consumption of Big Olaf brand ice cream [81]. Twenty-three cases of listeriosis were then noted, of which 22 were hospitalised (with one death noted). The summarised data indicate the need for the continuous monitoring of food of both animal and plant origin for the occurrence of *L. monocytogenes*.

**Table 2:** Selected *L. monocytogenes* outbreak infection cases around the world (2009–2022).

	Year (Period)	Country (City, State)	Source	No. of Cases	Reported Deaths	Reference
Europe	2009–2012	Portugal	Cheese	30	11	[64]
	2015–2018	Austria, Denmark, Finland, Sweden, the UK	Frozen corn	41	6	[66]
	2015	Denmark, Germany, France	RTE salmon products	12	4	[65]
	2014–2019	Denmark, Estonia, Finland, France, Sweden	Cold-smoked fish products	22	5	[67]
	2017–2019	Netherlands, Belgium	RTE meat products	21	3	[67]
	2019	Spain	Chilled roasted pork meat product	222	3	[68]
USA	2010–2015	Four states	Ice cream	10	3	[71]
	2014	Multi-states	Mung bean sprouts	5	2	[70]
	2014		Stone fruit	4	0	[83]
	2014–2015	Twelve states	Caramel apples	35	7	[69]
	2015–2016	Two states	Packaged leafy green salads	23	1	[84]
	2015	10	Soft cheeses	30	3	[75]
	2016	2	Raw milk	2	1	[73]
	2016	4	Frozen vegetables	9	3	[74]
	2016	9	Packaged salads	19	1	[72]
	2017	4	Soft, raw-milk cheese	8	2	[76]
	2019	5	Hard-boiled eggs	8	1	[77]
	2020	4	Deli meats	12	1	[79]
	2020	Seventeen states	Enoki mushroom	36	4	[78]
	2021	Two states	Frozen fully cooked chicken products	3	1	[80]
	2022	11	Ice cream	28	1	[81]
	2022	8	Packaged salads	10	1	[82]
Africa	2017–2018	Republic of South Africa	RTE processed meat products	1024	200	[85]
Australia	2018	Australia (New South Wales, Victoria, Queensland, Tasmania)	Rockmelons	20	7	[86]

Compiled from Wiktorczyk-Kapischke et al., (2023) [12] and Kaptchouang Tchatchouang et al., (2020) [87].

## 3. High-Pressure Processing—Potential Food Safety Risks

Nowadays, in response to increasing consumer demand for high-quality, ready-to-eat (RTE) products that are microbiologically safe while maintaining unchanged quality characteristics (nutritional and organoleptic value), the food industry is striving to find new food processing technologies aimed at producing products with minimally altered quality characteristics, a long shelf life, and no preservatives [88]. High-pressure processing is experiencing tremendous development in food preservation processes and is one of the most promising non-thermal methods of food preservation. HPP is a commercial technology used to extend the shelf life of many different types of food: liquid products, such as juices, fruit and vegetable purees, and guacamole; and meat products, including ready-to-eat sliced deli meat, hot dogs, dry-cured meat products, shellfish, ready-to-eat products, dips, wet salads, pureed baby foods, and dairy products [63]. Products preserved using this technology are required to be stored at a temperature below 7 °C in case of storage and distribution, with a shelf life (depending on the product) ranging from a few days to a few weeks [63,89]. It is one of the most promising non-thermal methods of food preservation that does not contribute to causing rapid changes in food degradation. Pascalisation also affects microorganisms—in the case of vegetative microorganisms, this technology is not able to meet all the challenges posed to it. Despite the recognition of pascalisation as an effective technology that produces safe, high-quality food, there are still many issues associated with the potential safety risks of using HPP [90]. These include the following issues:

- The control of surviving microbial spores (e.g., with refrigerated storage or additives) and HPP-induced spore activation (with subsequent conversion to vegetative cells);
- The induction of sub-lethal damage in cells, including the transformation of cells into a viable but non-culturable state (VBNC) (this can lead to the overestimation of HPP efficacy via routine detection methods);
- The induction of pathogenicity (virulence), gene expression (virulence, antibiotic resistance, and others), and cross-resistance to other stresses [91].

### 3.1. Determinants of the Effect of HPP Treatment on Listeria monocytogenes

*L. monocytogenes* exhibits resistance to many different environmental conditions [92]. In addition to inactivating pathogens found in food [93,94], the interactions that occur during food preservation using high pressures induce structural, morphological, physiological, and genetic changes, as well as damage to the pathogen cells directly [9]. The susceptibility of *L. monocytogenes* to HPP-induced stress factors, and thus the associated survivability of the pathogen, is determined by a number of different factors, of which the main ones include the HPP treatment parameters, namely the process temperature, pressure level applied, process duration, food matrix type, individual characteristics of strains, and their initial count (Table 3) [10,14,55,60,95,96,97,98,99,100,101,102,103,104,105,106,107,108,109,110,111,112,113]. Research results also point to synergistic interactions between HPP and other food preservation methods or the addition of substances with a beneficial bactericidal effect that may contribute to a more effective reduction in the *L. monocytogenes* population, even to microbiologically safe levels [60,103]. Differences have also been noted in the determination of *L. monocytogenes* count following HPP, depending on the growth medium used [100,102].

#### 3.1.1. HPP Treatment Parameters

This study indicates the effectiveness of different combinations of HPP treatment parameters in terms of the pressure (350–600 MPa), duration of treatment (1–20 min), and process temperature (4–40 °C), depending on the matrix/food, the initial *L. monocytogenes* population size, or the specific characteristics of the tested strain/cocktail of strains. The combination of these parameters/characteristics is important in terms of reducing the population to an appropriate level of safety. Researchers have indicated the lower pressure value and/or the duration of HPP treatment needed to inactivate *L. monocytogenes*, as compared to, e.g., *Escherichia coli* and *Staphylococcus aureus* [89]. With the same values of the duration of treatment and the temperature of the HPP treatment, increasing the pressure value tends to increase the level of reduction in the initial microbial count [114]. In most of the studies cited in Table 3, increasing the value of one of the HPP treatment parameters (pressure, duration of treatment, or process temperature) directly increased the reduction in the *L. monocytogenes* population to a microbiologically safe level (reduction > 5 log CFU/g (CFU/mL)), or ensured a level of reduction that prevented the detection of the pathogen via the culture methods employed in the study. In some of them, the level of reduction following an increase in one of the process parameters increased to a level that did not guarantee microbiological safety [95,97,99,102,103,112].

The literature provides information on the effectiveness of pressure parameters in relation to reductions in *L. monocytogenes*. In general, the application of a pressure > 400 MPa in most cases has the effect of reducing the initial population below the detection level, thus ensuring the microbiological safety of food (regardless of the time and temperature of the HPP treatment) (Table 3). 

The process duration has a significant effect on the effectiveness of HPP. Park et al., (2022) [60] analysed the effectiveness of HPP (500 MPa, 4 °C) against a cocktail of six *L. monocytogenes* strains in raw beef in three time variants (2, 5, and 7 min). The study showed an increase in the degree of reduction from 3.90 log CFU/g over 2 min to ≥6.50 log CFU/g over 7 min. An increase in the degree of reduction, dependent on the duration of HPP, was observed in a study by Stratakos et al., (2021) [105]. The researchers analysed the effect of HPP (400, 500, and 600 MPa; 18 °C) on the effectiveness of reducing a cocktail of five *L. monocytogenes* strains in raw milk over 1, 3, and 5 min. The researchers observed an increase in the degree of reduction with increasing storage time; however, this effect was not significant, as the reduction increased from 5.65 log CFU/g (3 min) to 5.91 log CFU/g (5 min). This study also observed that the effectiveness of the use of a pressure of 500 MPa and a duration of 5 min in the reduction in *L. monocytogen*es was similar to that when using parameters of 600 MPa/3 min. On the other hand, a study by Alpas and Bozoglu (2002) [96] observed different levels of reduction in a cocktail of five *L. monocytogenes* strains depending on the process temperature (25, 40, and 50 °C), with the ranges of 0.92–3.53, 8.20–8.70, and 7.78–8.04 CFU/mL, respectively (depending on the juice type used), which indicates the direct effect of the process temperature on the effectiveness of pathogen reduction.

The conditions under which the HPP treatment is carried out should be selected in such a manner so as to ensure the microbiological safety of food and thus not contribute to the deterioration of its quality characteristics. In certain cases, a positive reduction in the population to the desired safety-ensuring level resulted in a deterioration of the quality characteristics of the product. Batty et al., (2019) [104] analysed the effect of HPP technology on the survivability of *L. monocytogenes* with the simultaneous effect of this technology on the sensory characteristics of a Camembert-type cheese. Although the parameters applied in this study (450 MPa/10 min and 550 MPa/10 min) contributed to a reduction in *L. monocytogenes* > 5 log CFU/g, there was a deterioration in the quality and appearance of the product that would not be acceptable to consumers.

#### 3.1.2. Food Matrix

The type of food matrix also affects the effectiveness of the HPP treatment. As regards the properties of the test matrix, it was reported that certain food components (proteins, fats, carbohydrates, lipids) might have an effect in reducing the efficiency of HPP, which means that the results regarding the reduction in the microbial population in experiments carried out using growth mediums cannot be directly applied to the actual conditions occurring during food preservation [114].

In a study on cheeses by Evert-Arriagada et al., (2018) [102], the authors observed a reduction in *L. monocytogenes* count by approx. 5–6 log CFU/g following treatment at 500 MPa; however, at a lower treatment (300 MPa), the resulting reduction amounted to only 0.7 log CFU/g. Similar results were obtained by Hnosko et al., (2012) [115] and Tomasula et al., (2014) [99] in the “Queso Fresco” cheese. Tomasula et al., (2014) [99] observed that the HPP treatment under a pressure of 600 MPa for 20 min at 20 °C was effective at reducing in the population of a cocktail of five *L. monocytogenes* strains inoculated on the surface or in the curd of Queso Fresco cheese slices. The potential for regeneration and growth was observed after 7 and 28 days, respectively. The authors believe that microstructural changes in the cheese matrix, induced by HPP, triggered the gradual aggregation of the protein matrix, accompanied by a loss of whey, which increased the resistance of *L. monocytogenes* to HPP in a manner similar to that of low water activity [116,117]. Some authors attributed these results to the level of fat used in cheese production due to its baroprotective effect [118]. Similar microstructural changes in a cheese matrix induced by HPP were previously described for a fresh cheese subjected to a pressure of 500 MPa for 5 min under industrial conditions [119].

The effectiveness of HPP is also determined by the type (consistency) of the product. A study conducted by Misiou et al., (2018) [103] to assess, e.g., the survivability of *L. monocytogenes* strains in three different food matrices critical to this microorganism (UHT milk, mozzarella cheese, smoked salmon) indicated a reduction to a safe level of the microorganism occurrence in all food matrices except smoked salmon, ensuring a reduction by only 1.50 log CFU/g (500 MPa, 10 min, 25 °C), while in the other matrices (with the same HPP treatment parameters), the population of *L. monocytogenes* decreased to a level below detection via the methods applied. 

The choice of growth medium for carrying out experiments has an effect on the effectiveness of reduction under the influence of HPP (Table 3). More enriched mediums, such as TSBYE (Tryptic soya broth with 0.6% yeast extract), may have a diminished effect on the reduction in the *L. monocytogenes* population than TSB (Tryptic soya broth) alone or BHI (Brain-heart infusion). At pressures >400 MPa, differences are observed in the population reduction level due to the fact that enriched mediums such as TSBYE will allow a higher initial level of *L. monocytogenes* to be obtained, and it is well known that the effectiveness of HPP is also determined by the initial pathogen occurrence level [14]. For this reason, it is important to consider the effect of the specific matrix on the effectiveness of the HPP treatment in the planned research.

#### 3.1.3. Individual Characteristics of Strains

The effectiveness of HPP treatment is determined by numerous properties, including the individual characteristics of test strains. Perez-Baltar et al., (2021) [112] investigated the survivability of two selected *L. monocytogenes* strains following exposure to HPP in sliced dry-cured ham and during storage in a refrigerator. The *L. monocytogenes* strains S2 and S7-2 exhibited moderate resistance to HPP (450 MPa for 10 min—a reduction by 0.80 log CFU/g; 600 MPa for 5 min—a reduction by 1.30 and 1.50 log CFU/g, respectively) in a sliced dried ham with a low a_w_ value of 0.88. Brusch et al., (2017) [100], similar to the few previously conducted studies, used a greater set of strains, i.e., 14 *L. monocytogenes* strains. The strains were isolated from food and of clinical origin and characterised by different phenotypic and genetic features. The authors of the study also observed a considerable intra-strain variability in pressure resistance among the tested strains. Overall, all the strains were able to survive treatments under pressures of 300, 400, and 500 MPa, with a loss of viability (in log cycles) ranging from 0.00 to 2.76, from 0.06 to 6.31, and from 0.75 to 7.23, respectively. The application of a pressure of 500 MPa was sufficient to reduce the viability of all the strains by more than five log cycles, except a single strain isolated from a fermented sausage (a reduction by <1.00 log CFU/g). The researchers also observed that antibiotic-resistant *L. monocytogenes* strains exhibited a higher survivability level following the application of a pressure of 400 MPa [100].

The exposure of *L. monocytogenes* strains representing different phenotypes and genotypes to different parameters and the food matrix indicates a high variability of their survivability. The use of *L. monocytogenes* strains exhibiting different resistance to pressure in the study is crucial in guaranteeing that the number of cells in food products subjected to HPP will decrease to an appropriate level, as well as the indication of optimal process parameters in order to ensure adequate food safety [100,102].

#### 3.1.4. Addition of Antibacterial Agents

In order to increase the effectiveness of HPP treatment, it is possible to use antimicrobial agents or essential oils in combination with HPP. Depending on the antimicrobial agent used, the synergistic effect may be either low or high. The literature provides a few reports indicating the possibility of a synergistic effect of HPP technology along with other methods contributing to a reduction in *L. monocytogenes* immediately following the treatment, and those contributing to the impairment of the pathogen’s regenerability during storage [98,100,103,106,111].

Certain studies focused on the addition of a substance/strain with documented anti-listeria properties [98,100,103]. A study conducted by Bruschi et al., (2017) [100] showed that the exposure of *L. monocytogenes* strains representing different phenotypes and genotypes to different hydrostatic pressures demonstrated a high variability in their survivability. This study also investigated a combination of HPP preservation with an anti-listeria pediocin bacHA-6111-2 produced by *Pediococcus acidilactici* HA-6111-2. The study showed that this bacteriocin could be used as a natural agent to prevent the reversal of cellular damage during the storage of products subjected to HPP. On the other hand, in a study by Misiou et al., (2018) [103], the authors investigated the effectiveness of a combination of endolysin PlyP825 and HPP against a cocktail of *L. monocytogenes* strains in selected food products (i.e., milk, mozzarella cheese, and smoked salmon). The authors demonstrated that the effectiveness of the combination of these methods was determined by the food type, yet it showed greater effectiveness than HPP alone. This study indicated that the application of PlyP825 enabled the elimination of several different *L. monocytogenes* strains from food at reduced levels of the HPP pressures applied. Patterson et al. (2011) [98] investigated the synergistic effect of HPP in combination with the pressure-resistant *Weissella viridescens* strain and HPP in combination with sodium lactate on the survival and regenerability of *L. monocytogenes* during storage. In their study, the researchers demonstrated the ability to reduce cell growth as a result of the addition of a *W. viridescens* strain (extension of the detection limit from 7 days to 21 days). Sodium lactate alone did not significantly inhibit the growth of *L. monocytogenes*; however, in combination with HPP, it proved to be the most effective of all the tested agents in inhibiting the pathogen’s growth, even during prolonged storage at 8 °C (below the detection level up to day 105 of storage).

The application of coatings and the combination of a few different preservation methods have also yielded satisfactory results in reducing the *L. monocytogenes* population. Bambace et al., (2021) [106] investigated the effect of HPP on reducing *L. monocytogenes* in combination with the application of an alginate–vanillin coating in ready-to-eat apple cubes. The study observed a decrease in the *L. monocytogenes* cell count below the detection level in each case (following HPP and following a combination of HPP and the coating applied). Interestingly, in the case of *E. coli*, the application of a coating increased the level of population reduction, as compared to the use of HPP treatment alone (from 8.02 log CFU/g to 2.61 log CFU/g). On the other hand, a study by Jarzynka et al., (2021) [111] assessed the potential for the growth of such pathogens as *L. monocytogenes* following the application of two methods combined, i.e., HPP and freeze-drying, for the preservation of donor milk. The authors report that both HPP alone and the combination of these two methods reduced the *L. monocytogenes* count below the detection level (a reduction of 7.91 log CFU/mL), even after six months of storage.

### 3.2. Recovery of Cells during Storage after HPP

The control of pathogenic bacteria is possible thanks to advances in the development of antimicrobial agents and the provision of proper hygienic conditions during food production. However, this “fight” continues due to the adaptability of microorganisms to changing environmental conditions as a result of intra-strain and intra-species transformations, leading to their production of certain defence mechanisms. One such mechanism which is receiving increasing attention is the ability of cells to enter a “dormant” state related to a “viable but not-culturable” (VBNC) state [120]. The VBNC state is one of the survival strategies adopted by many microbial species when exposed to extreme environmental stress conditions. This state resembles microbial “dormancy”, as these cells are not able to grow on solid growth mediums while retaining their metabolic activity, expression of toxic proteins, and increased tolerance to antimicrobial agents (including antibiotics) [120,121]. The inability to use the commonly employed culture methods to determine cells in the VBNC state is a major impediment in experimental studies concerning, e.g., increased antibiotic resistance, and hinders the effective assessment of the control of cells in this state [120]. Currently, more than 100 microbial species are able to enter the VBNC state. There are scientific reports in the literature relating to the fact that cells in the VBNC state can produce biological toxins (through the presence of toxin-encoding genes). Cells in this state may show a higher expression of specific genes as compared to cultured cells [122,123]. In addition, cells in the VBNC state can return to a viable state under favourable environmental conditions. All the above-mentioned arguments indicate that pathogens in the VBNC state pose a serious hazard to the microbiological safety of food and, consequently, to human health [20]. VBNC cells have different morphological characteristics than cultured cells, as the cross-linking of peptidoglycan in the cell wall is denser, the composition of lipids in the cytoplasmatic membranes is different, and metabolism is reduced, allowing them to remain in this state for a long time, even for several months. They are able to retain their pathogenic potential despite the considerably reduced metabolism and, under favourable conditions, can revert to vegetative forms while retaining an increased pathogenicity level [124].

A few previously conducted studies showed the presence of sub-lethally damaged *L. monocytogenes* cells capable of regenerating following HPP and growing during storage (even under refrigeration) [13,99,101,108,110,113,125,126,127]. In certain studies, immediately after HPP, no presence of *L. monocytogenes* cells was noted. However, with increasing storage time, the cells regenerated and grew, which suggests possibilities relating to the transition of *L. monocytogenes* to the VBNC state. These observations were made following the application of HPP in many combinations of pressure (200–600 MPa), time (3–30 min), temperature (4–45 °C), and the storage duration and temperature (14–75 days; 4–37 °C) for both food products and growth mediums (Table 4).

#### 3.2.1. Cell Recovery in the Food Matrix

A study by Tomasula et al., (2014) [99] showed the presence of damage to the surface of *L. monocytogenes* cells in a packaged Queso Fresco (QF) cheese, using scanning microscopy (at 5000× magnification), following HPP treatment under pressures of 400 and 600 MPa, with the changes being more pronounced at a higher-pressure values (visible bud scars and cell shrinkage). Before the HPP treatment, the surfaces of the *L. monocytogenes* cells were smooth, similar to their state after the treatment with a pressure of 200 MPa. Increasing the 200 MPa pressure action time to 20 min resulted in the formation of bud scars on the surface of the cells, which indicated that the membrane was disrupted or lost its integrity. Even though the disruption was visible, it did not cause cell death. The study found that, irrespective of the HPP treatment parameters, most of the *L. monocytogenes* cells (at 4 °C) were damaged, and a decrease was noted in the percentage of damaged cells as the storage time progressed (up to day 14). Compared to a temperature of 10 °C, a small, statistically insignificant change was observed in the difference in the *L. monocytogenes* count (a higher temperature facilitated the growth of the pathogen). In general, the exposure of QF to the HPP treatment (600 MPa, 3 min) resulted in the regeneration of damaged cells and the growth of *L. monocytogenes* within 1.00 log CFU/g after 42 days of storage, to reach a value of 8.24 ± 0.41 log CFU/g after 60 days of storage. A study by Valdramidis et al. (2015) [108] concerning the assessment of pressurisation conditions in the production of meat products with reduced salt content and their effect on the development of *L. monocytogenes* during storage observed that all the pressurisation treatments within a range above 500 MPa significantly reduced the *L. monocytogenes* count (below the detection level immediately after treatment). However, over the subsequent days of storage, the growth of *L. monocytogenes* in the tested samples was observed. A pressure of approx. 450 MPa had no significant effect on the levels of *L. monocytogenes* immediately after treatment or during storage. Only a combination of pressures of 700 and 800 MPa with different salt concentrations resulted in the preserved product being microbiologically stable even after a 28-day storage period. The regenerability of *L. monocytogenes* cells, similar to that showed in the cited results, was also observed by Jofré et al. (2010) [110]—the researchers demonstrated the possible growth of *L. monocytogenes* cells in broth mediums following the treatment with pressures of 400 and 600 MPa for 10 min; even if immediately following HPP, the number of cells was below the detection level.

#### 3.2.2. Cell Recovery in Growth Mediums

A study by Bozoglu et al., (2004) [13] showed that no *L. monocytogenes* cells could be detected immediately after the treatment with pressures of up to 550 MPa. However, the same study demonstrated the presence of damaged but viable *L. monocytogenes* cells in pressure-treated samples that were able to grow after six days of storage at 4 °C, as well as after one day at 22 °C and 30 °C, on both a selective and non-selective growth medium, which suggests the existence of secondary damage. Nakaura et al., (2019) [127] proved that the regeneration of HPP-damaged *L. monocytogenes* cells on growth mediums (TSB and PBS) was determined by many factors, from the availability of nutrients to the duration and temperature of storage. The researchers demonstrated that most of the cells that were damaged following pressurisation (500 MPa, 25 °C, 10 min) during a 42-day period of storage at 5 °C, 10 °C, and 15 °C regained their ability to proliferate and were able to reach the initial count or even exceed it. This study demonstrated that the regeneration of *L. monocytogenes* cells subjected to the HPP treatment at 0 °C was limited in comparison with 5 °C, 10 °C, or 15 °C, and was more intense at temperatures lower than the optimal bacterial growth temperature. These results are consistent with the results presented in other publications [125,126]. A study by Bull et al., (2005) [125] proved the ability of *L. monocytogenes* cells to regenerate during storage after treatment with pressures of 450 and 600 MPa, and differences in the regeneration of pathogen cells, resulting from the type of medium used, temperature, and storage duration. The study demonstrated that damaged *L. monocytogenes* cells could regenerate in specially enriched mediums after milk samples were stored at 4 °C, 15 °C, and 30 °C (maximum regeneration after 24–72 h of storage), with the regeneration efficiency decreasing to 0% after the longer storage of milk at 4 °C and 30 °C. The study also noted that storage at 15 °C affected the highest regeneration rate (regeneration efficiency at a level of 100%), which remained high for 14 days of storage. On the other hand, a study by Koseki and Yamamoto (2007) [126] investigated the effect of various combinations of pressure (200–400 MPa), durations of treatment (1–30 min), and growth mediums on the regenerability of cells following storage at different temperatures (20 °C and 37 °C) and storage times (10, 20, and 30 days). It was found that the pathogen cells were able to regenerate, even if no viable cells were detected immediately after HPP, following storage for 10–30 days at 20 °C (no regeneration of cells was detected during storage at 37 °C).

The cited studies clearly indicate that HPP (in various combinations of pressure, duration of treatment, and temperature) can induce not so much the death of *L. monocytogenes* cells, but rather their damage and transformation to a VBNC state. Due to the regenerability of *L. monocytogenes* cells during storage (even under refrigerated conditions and a storage period of ≥42 days from the time of HPP treatment), they exhibit regenerability and the possibility to return to viability, which may consequently translate into a return of the population to a level comparable with its initial count [99,127]. These properties are clearly determined by many variables (e.g., parameters, the type of matrix used, storage temperature, and duration), which should be taken into account when carrying out subsequent studies [108,113,127].

### 3.3. Effect of HPP on Antibiotic Resistance and Antibiotic Resistance Gene Expression

The environmental stress induced by the effects of food preservation methods undoubtedly has a significant impact on changes in the antibiotic resistance of microbial strains. The scientific literature provides little information on the effect of pascalisation on antibiotic resistance and the expression of genes encoding this resistance in *L. monocytogenes* strains. Only a few reviews have focused on investigating the impact related to strain tolerance to pascalisation and antibiotic resistance [10,100].

The first study concerning an assessment of the survivability of antibiotic-resistant *L. monocytogenes* strains following HPP treatment was described by Bruschi et al. (2017) [100]. The study used strains resistant to one or two antibiotics simultaneously (tetracyclines, ciprofloxacin, erythromycin, nitrofurantoin). The researchers showed that antibiotic-resistant *L. monocytogenes* strains exhibited higher survivability rates following the application of HPP at a pressure value of 400 MPa.

Duru et al., (2020) [10] in their study examined the relationship between the presence of antibiotic resistance genes and tolerance to HPP-induced stress. The obtained data on gene occurrence did not indicate the existence of antibiotic resistance-encoding genes specific to the strains under analysis. The same genes encoding antibiotic resistance were detected in all *L. monocytogenes* strains that were analysed (*n* = 10) (*FosX*, *mprF*, *norB*, *lin*). For the first three genes, differences in amino acid sequences between strains were demonstrated. However, it is not known whether these differences contribute to an increase in antibiotic resistance. The researchers also demonstrated that the amino acid sequence of the gene encoding resistance to quinolones (*norB*) and of the gene encoding resistance to lincomycin (*lin*) differed slightly between strains. Interestingly, the differences in gene expression were related to the time following the action of pressure and to the strain type. They thus confirmed the observation that differences in antibiotic resistance genes may ensure different levels of barotolerance within the *L. monocytogenes* strains. The researchers believe that it is actually possible to use antibiotic resistance more effectively in protection against pressurisation-induced stress. 

### 3.4. Effect of HPP on Virulence Factors and Expression of Virulence Genes

*L. monocytogenes* possess a number of defence mechanisms against the adverse environmental conditions prevailing during food processing that enable adaptation and/or survival [128]. One of the mechanisms is the ability of cells to exchange information (i.e., communicate with each other). One of the most important microbial communication systems that transforms individual behaviours into group ones is the *quorum sensing* (QS) system that uses signalling molecules, so-called autoinducers. Autoinducer-2 (AI-2) is a signalling molecule produced by the LuxS enzyme that allows bacteria to organise into a biofilm that provides an ideal environment for pathogen growth and development, which is a key concern for the food industry. This enzyme is also a key enzyme in the activated methyl cycle, a crucial central metabolic pathway [129,130]. 

The impact of sub-lethal stresses leads to the adaptation of *L. monocytogenes* to unfavourable environmental conditions, which consequently translates into changes in the expression profiles of specific sets of genes [131]. The potential of *L. monocytogenes* to respond to adverse conditions or changes in the environment is determined by various alternative sigma SigB (σ^B^) factors and the genes it controls, which contribute to an increase in this pathogen’s survival of processes such as HPP [132]. This factor is responsible for inducing the transcription factor PrfA (the main virulence regulator of *L. monocytogenes*) and, therefore, plays a very important role in the virulence of this pathogen [128,133,134]. The alternative sigma factor (SigB (σ^B^)) allows *L. monocytogenes* to proliferate and survive under stressful conditions in non-host environments [135], including those found in foods, such as acidic or osmotic conditions [132,136].

HPP-induced environmental stress can directly contribute to the activation of virulence mechanisms and the expression of specific sets of genes involved in virulence. Currently, there are few scientific reports on the effect of environmental stress on the expression and regulation of virulence genes in *L. monocytogenes* that survive HPP treatment in food matrices [112,128] and growth mediums [14,15,137] (Table 5).

Previous research has focused on the assessment of the expression of the following genes involved in virulence: *plcA*, *hly*, *iap*, *sigB*, *prfA*, *luxS*, *hpf*. A study by Pérez-Baltar et al., (2020) [112] assessed the expression of three virulence genes (*plcA*, *hly*, *iap*) and one stress-related gene (*sigB*) in a dried ham with different water activities (a_w_) (0.92, 0.88, 0.84) under the influence of pressurisation (450 MPa/10 min and 600 MPa/5 min) over 30 days of storage at 4 °C in two *L. monocytogenes* strains representing different serotypes, namely serotype 1/2b (S4-2) and serotype 1/2c (S12-1). The researchers concluded that the HPP-surviving bacteria gene transcription patterns were strain-dependent. In the strain belonging to serotype 1/2b, the *plcA*, *hly*, *iap* and *sigB* genes were overexpressed, while in the strain belonging to serotype 1/2c, the analysed genes were suppressed. The induction of both overexpression and suppression of the analysed genes was lower for samples with the lowest a_w_. Such differences had already been previously observed [138,139]. According to the authors, the greater damage caused by the greater susceptibility of the strain belonging to serotype 1/2c to HPP resulted in increased expression of the genes responsible for cell regeneration without the activation of the genes associated with virulence and stress response. Another study, carried out by Pérez-Baltar et al., (2021) [128], evaluated the effect of HPP on the expression of five genes involved in virulence and stress response of two *L. monocytogenes* species representing different serotypes, i.e., S2 (serotype 1/2a) and S7-2 (serotype 4b). The researchers noted suppression of the expression of genes *sigB* and *prfA* immediately following the application of pressure of either 450 or 600 MPa for the first of the analysed strains. For the second strain, the relative expression of both analysed genes was significantly upregulated (*p* < 0.05), being more pronounced at 450 MPa. When stored at 4 °C, gene overexpression was suppressed (a significant reduction from day 7 onwards). As for the *plcA* and *hly* genes, immediately after treatments, a slightly repressed expression was noted for the first analysed strain and overexpression for the second one. As regards the *plcA* gene, the transcription level was lower than that for *hly*.

Some studies focused on the analysis of genes involved in virulence in the growth medium. Bowman et al., (2008) [137] reported suppression of the expression of the genes associated with cell growth and virulence when *L. monocytogenes* strains were subjected to high pressures in TSYE broth. Treatment with a pressure of 400 or 600 MPa for 5 min resulted in a decrease in *sigB* and *prfA* gene expression for the *L. monocytogenes* strain belonging to serotype 1/2a, with the pressure of 400 MPa causing a greater decrease in expression. This study also observed an increase in the expression of genes associated with motility and chemotaxis, which can be linked to both the ongoing repair processes and changes in the energy capacity of the cell. A study by Chen et al., (2021) [15] showed a significant increase in the expression of *LuxS* following the HPP treatment, which may consequently lead to the promotion of the activated methyl cycle, quorum sensing, and biofilm formation. In addition, the researchers noted differences in the expression of genes involved in numerous other processes, including cell cycle control, cell division, and chromosome partitioning; cell wall, membrane, and envelope biogenesis; energy production and conversion; lipid transport and metabolism; nucleotide transport and metabolism; post-translational modification, protein turnover and chaperone; replication, recombination and repair; transcription as well as translation, ribosomal structure and biogenesis. A study by Duru et al., (2021) [14] analysed the expression of genes during the regeneration of *L. monocytogenes* at different time intervals following HPP treatment at two different pressure values (200 MPa and 400 MPa). The experiments were conducted using two strains, i.e., RO15 (line II, serotype 1/2a), a strain more resistant to HPP than the other ones, and ScottA (line I, serotype 4b), a strain more susceptible to HPP. The researchers showed that the stress response was activated by the general stress factor B (σ^B^). The mainly affected the expression of genes responsible for protein folding, PTS system genes (phosphotransferase system; mostly fructose-, mannose-, galactitol-, cellobiose-, and ascorbate-specific PTS systems) and cobalamin biosynthesis genes. The genes mentioned earlier were the most upregulated genes during HPP damage recovery. The researchers observed that cell-division-related genes (*divIC*, *dicIVA*, *ftsE*, and *ftsX*) were downregulated. By contrast, peptidoglycan-synthesis genes (*murG*, *murC*, and *pbp2A*) were upregulated, indicating regeneration of cell damage after HPP through cell wall repair. The researchers also observed that the non-encoding RNA *Rli47* plays a role in the regeneration of post-HPP damage in *L. monocytogenes*, and that the *pbp2A* mutants were more susceptible to HPP.

## 4. Conclusions

The cited study results indicate relationships between different combinations of pressure, duration of treatment, temperature, type of matrix used, individual strain characteristics, developmental stage or storage parameters, and survivability, antibiotic resistance, virulence, and gene expression in *L. monocytogenes* strains under the influence of HPP-induced environmental stress. The literature review presents the current knowledge on the effects of HPP on survivability, regenerability of the cells after storage, virulence, and antibiotic resistance of *L. monocytogenes*. It also points to the lack of sufficient knowledge on the assessment of antibiotic resistance, virulence and the expression of antibiotic resistance genes and virulence in the *L. monocytogenes* cells that survived. The available reports are only an indication towards future research directions. More strains need to be evaluated to explain which individual strain characteristics are associated with the HPP-induced cellular response. It is also necessary to assess the changes in antibiotic resistance and virulence of *L. monocytogenes* strains.

## Figures and Tables

**Table 1:** *Listeria monocytogenes* virulence factors and their function.

Virulence Factors		Protein/Gene	Function
Protein Regulatory Factor		PrfA	Regulator of expression of many virulence proteins
Sigma B		σ^B^	Regulation of stress and virulence genes
**Adhesion Proteins**	*Listeria* adhesion protein	LAP	Adhesion to intestinal epithelial cells; disruption of intestinal epithelial barrier
	*Listeria* adhesion protein B	LapB	Adhesion and invasion into host cells
	Autolysin amidase	Ami	Adhesion to hepatocytes
	Fibronectin binding protein	FbpA	Adhesion to cells and serves as a chaperone to stabilise and secrete LLO, InlB
	Internalin J	InlJ	Adhesion to epithelial cells and binds to human intestinal mucin-2 (MUC2)
	Internalin F	InlF	The crossing of blood–brain barrier
	Autolysin IspC	-	Adhesion to non-phagocytic cells
	Lmo1656	-	Transcytosis in goblet cells
**Invasion**	Internalin	InlA	Promotes bacterial internalisation into enterocytes and bacterial transcytosis across the intestinal barrier
	Internalin B	InlB	Acts in the invasion of enterocytes and passage through M-cells of Peyer’s patches
	Virulence invasion protein	Vip	Invasion of epithelial cells
	LAP	-	Induces junctional protein dysregulation and increases epithelial permeability (translocation)
**Lysis of vacuole**	Listeriolysin (LLO)	*hlyA*	A haemolysin helps bacteria escape from the phagosome inside the cell by disrupting the vacuolar membrane
	Phospholipase	(*plcA*–PI-PLC; *plcB*–PC-PLC)	Lyses of vacuole membrane
**Cell-to-cell spread**	Actin polymerisation protein	ActA	Nucleation of actin tail for bacterial movement inside the cytoplasm
	PC-PLC	-	Lyses of vacuole membrane
	Metalloprotease	Mpl	Helps synthesis of PLC
**Miscellaneous**	P60 (cell wall hydrolase)	-	Adhesion/invasion
	Bile salt hydrolase	BSH	Survival in gut
	Fructose-1,6- bisphosphate aldolase	FAB	Moonlighting protein: (i) adhesion to the host’s cells and (ii) role in the pathogenesis
	Internalin C	InlC	Perturbs apical cell junctions
	Internalin H (InlH)	InlH	Contributes to systemic listeriosis
	Autolysin amidase	Ami	Bacteriolysin: enhances the host immune response
	LAP	-	Upregulates TNF-a and IL-6 expression in intestinal cells
	Listeriolysin (LLO)	*hlyA*	Induces lymphocyte apoptosis and suppresses proinflammatory cytokines
	Listeria nuclear-targeted protein A	IntA	Decreases the host’s immune response
	Listeriolysin S	-	Haemolytic and cytotoxic; bacteriocin (bactericidal)

Compiled from Bhunia (2018) [27] and Lopes-Luz et al., (2021) [28].

**Table 3:** Reduction in *L. monocytogenes* populations by HPP in food, and growth mediums.

No.	Strains	HPP			Reduction in Population (log CFU/g or log CFU/mL)	Food/Medium	Reference
		Pressure (MPa)	Time (min)	Temp. (°C)			
1	One strain	100	3	12	NE	Tryptic soy broth (TSB)	[55]
		200					
		300			1.49		
		400			BD		
2	One strain	200	10	20	NE	pH 5.6 citrate buffer	[95]
		300					
		400			BD		
3	Cocktail of 5 strains	200	5, 10, 15, 20	20, 40	NE	Queso Fresco (QF) cheese	[99]
		400			~1.78-BD		
		600			BD		
4	Cocktail of 2 strains	200	8	8	NE	TSBYE	[14]
		400			5.78 and 7.04		
5	Cocktail of 5 strains	200	10	25	NE (I, II, III)	UHT milk (I) Mozzarella (II) Smoked salmon (III)	[103]
		300					
		400			3.00–4.00 (I, II); NE (III)		
		500			BD (I, II) 1.50 (III)		
6	Cocktail of 9 strains	250	5	30	3.90–4.34	Pasteurized fruit juices (apple, apricot, cherry, and orange)	[96]
		350		25	0.92–3.53		
				40	8.20–8.70		
				50	7.78–8.04		
7	Cocktail of 14 strains	300	5	10	0.00–2.76	TSBYE	[100]
		400			0.06–6.31		
		500			0.75–7.23		
8	Two strains	300	10	5, 20	NE	Cheeses	[97]
		400			2.97 and 1.57		
		500			5.00		
9	Two strains	300	5	6	NE	Commercial free-starter fresh cheese	[102]
		400					
		500			BD ^−^ and BD ^+^ 1.50 ^−^ and 2.00 ^+^		
		600			BD ^−^ and BD ^+^ 3.90 ^−^ and 4.30 ^+^		
10	Cocktail of 4 strains	350	10	25	~2.00	Camembert cheese	[104]
		450			>5.00		
		550					
11	One strain	400	5	35	>5.00	Apple cubes	[106]
12	Cocktail of 10 strains	400	1	20	0.05–2.07	TSBYE	[10]
		600			5.42–8.27		
13	Cocktail of 5 strains	400–800	3	20	2.00-BD	Meat simulation medium	[108]
14	One strain	400	15	20	4.00 ^−^ and 6.70 ^+^	UHT milk	[109]
		500					
		550			4.00 ^−^ and 7.00 ^+^		
		600					
15	Cocktail of 5 strains	400	1, 3, 5	18	1.42 (1 min)	Raw milk	[105]
		500			5.48 (5 min)		
		600			5.65 (3 min) 5.91 (5 min)		
16	Cocktail of 5 strains	400	10	15	≥8.00	Brain-heart infusion (BHI)	[110]
		600					
		900	5				
17	One strain	450	15	21	≥7.91	Human milk	[111]
18	Cocktail of 2 strains	450	10	16	0.80	Sliced dry ham	[112]
		600	5		1.30 and 1.50		
19	Cocktail of 7 strains	593	3	4	≥6.00	Coconut water	[113]
20	Cocktail of 5 strains	500	2, 5, 7	4	3.90 (2 min) ≥6.50 (7 min)	Raw beef	[60]
21	Cocktail of 4 strains	600	8	16	2.47 (DCS); 2.13 (DCL)	Dry-cured salchichón (DCS) and dry-cured loin (DCL)	[107]
22	Cocktail of 13 strains	600	2	20	5.64-BD	Cooked chicken	[98]
23	Cocktail of 4 strains	600	3	4	≥7.50	Cooked pork sausage	[101]

NE—treatment not enough to reduce the population of *L. monocytogenes* strains; BD—reduction in the *L. monocytogenes* population to below detection levels; ^−^—initial inoculation value at the level of 3–4 log CFU/g; ^+^—initial inoculation value at the level of 6–7 log CFU/g; TSBYE—Tryptic soy broth with 0.6% *w*/*v* yeast extract.

**Table 4:** Possibilities of recovery of *L. monocytogenes* after HPP in food, and growth mediums.

No.	Strains	HPP			Storage Analyses			Food/Medium	Reference
		Pressure (MPa)	Time (min)	Temp. (°C)	Time (Days)	Temp. (°C)	Recovery/Presence of Damaged Cells		
1	Cocktail of 5 strains	200	5, 10, 15, 20	20, 40	60 (0, 7, 14, 28, 42, 60) *	4, 10	(+) ≥42 day recovery	Queso Fresco cheese (QF)	[99]
		400							
		600							
2	One strain	200–500	1, 3, 5, 10, 20, 30	22 ± 2	30 (10, 20, 30) *	20	(+)	Medium	[126]
						37	(–)		
3	One strain	350	10	45	28 (0, 2, 4, 6, 8, 10, 12, 14, 17, 20, 26, 28) *	4	>6 day (+)	UHT milk	[13]
		450				22	>1 day (+)		
		550				30			
4	Cocktail of 5 strains	400	10	15	21 (2, 7, 21) *	14	(+) >21 days more recovery	Brain-heart infusion (BHI)	[110]
		600							
		900	5			22	>2 day (+)		
5	Cocktail of 5 strains	400–800	3	20	28 (7 days intervals) *	8	≥700 MPa (–)	Meat simulation medium	[108]
6	Cocktail of 5 strains	450	15	18 ± 2	14 (daily) *	4	>72 h (–)	Skim/whole raw milk	[125]
		600	1.5			15	≤14 days (–)		
						30	>72 h (–)		
7	One strain	500	10	25	42 (daily) *	0	(–/+)	Trypticase soy broth (TSB) and phosphate-buffered saline (PBS)	[127]
						5	(+)		
						10			
						15			
8	Cocktail of 7 strains	593	3	4	75 (1, 7, 14, 28, 45, 60, 75) *	4	(–)	Coconut water	[113]
						10			
9	Cocktail of 4 strains	600	3	4	35 (0, 7, 14, 21, 28, 35) *	4	(–)	Cooked pork sausage	[101]
						10	≤21 day (–) 35 day (+)		

*—days of sampling for testing; (–)—no presence/no recovery during the whole storage period; (+)—presence/recovery during the storage period (–/+)—limited recovery during the storage period.

**Table 5:** Effects of HPP on the expression of genes encoding *L. monocytogenes* virulence.

No.	Strains	HPP			Virulence Genes	Expression	Food/Medium	Reference
		Pressure (MPa)	Time (min)	Temp. (°C)				
1	2 strains	450	10	19	*plcA*, *hly*, *iap*, *sigB*	General strain-dependent overexpression/suppression (mainly *hly sigB*, *plcA*)	Dry-cured ham	[112]
		600	5					
2	2 strains	450	10	16	*prfA*, *plcA*, *hly*, *sigB*, *lmo1421*			[128]
		600	5					
3	1 strain	200	3	12	*luxS*	Upregulation	Trypticase soy broth (TSB)	[15]
		400						
4	2 strains	200	8	8	*sigB*, *hpf*, *prfA*	Upregulation	TSBYE	[14]
		400						
5	2 strains	400	5	15	*sigB*, *prfA*	Strong suppression	TSBYE	[137]
		600						

TSBYE—Tryptic soy broth with 0.6% *w*/*v* yeast extract.

## Data Availability

Data are contained within the article.
